# Dietary ecological momentary assessments: validation against food diaries and associations with health biomarkers

**DOI:** 10.3389/fnut.2026.1927498

**Published:** 2026-09-04

**Authors:** Anna Boronat, Natalia Soldevila-Domenech, Joana Clivillé-Pérez, Aida Cuenca-Royo, Maria Gomis-González, Thais Lorenzo, Iris Piera, Klaus Langohr, Montserrat Fitó, Helmut Schröder, Oriol Grau-Rivera, Nieves Pizarro, Laura Forcano, Rafael de la Torre

**Affiliations:** 1 Integrative Pharmacology and Clinical Neurosciences, Hospital del Mar Research Institute, Barcelona, Spain; 2 Clinical Research and Risk Factors for Neurodegenerative Diseases Group, Barcelonaβeta Brain Research Center, Pasqual Maragall Foundation, Barcelona, Spain; 3 Department of General and Digestive Surgery, Hospital del Mar, Barcelona, Spain; 4 Departament d’Estadística i Investigació Operativa, Universitat Politècnica de Catalunya—BarcelonaTech, Barcelona, Spain; 5 Grup de recerca en Risc Cardiovascular i Nutrició, Hospital del Mar Research Institute, Barcelona, Spain; 6 Centro de Investigación Biomédica en Red de Obesidad y Nutrición (CIBEROBN), Madrid, Spain; 7 Grup de recerca en Epidemiologia i Genètica Cardiovascular, Hospital del Mar Research Institute, Barcelona, Spain; 8 Centro de Investigación Biomédica en Red de Epidemiologia y Salud Pública (CIBERESP), Madrid, Spain; 9 Centro de Investigación Biomédica en Red de Enfermedades Neurodegenerativas (CIBERNED), Madrid, Spain; 10 Medical and Life Sciences (MELIS) Department, Universitat Pompeu Fabra, Barcelona, Spain

**Keywords:** convergent validity, dietary monitoring, digital dietary assessment, ecological momentary assessment, Mediterranean diet adherence, mobile health, nutritional intervention, real-world assessment

## Abstract

**Background:**

Digital approaches that capture dietary behavior repeatedly in real-world settings may improve the monitoring of long-term lifestyle interventions while reducing participant burden and supporting self-awareness. This study developed and evaluated a low-burden dietary ecological momentary assessment (EMA) protocol to monitor Mediterranean diet adherence during the 12-month PENSA multimodal lifestyle trial in older adults at increased risk of cognitive decline.

**Methods:**

A total of 104 participants aged 60–80 years with subjective cognitive decline and APOE-ε4 carrier status were included. Daily mobile EMA prompts assessed key Mediterranean diet food groups through a rotating schedule, and participants received personalized feedback every 4 weeks. EMA-derived adherence indicators were evaluated against the Mediterranean Diet Adherence Screener, 3-day food diaries, nutrient profiles, usability data, and cardiometabolic and dementia-risk markers.

**Results:**

Mediterranean diet adherence improved over the intervention, with EMA scores increasing from 73.6 to 82.3% and capturing dynamic changes in dietary quality across the year. EMA-derived indicators showed increasing convergence with the Mediterranean Diet Adherence Screener over time (*ρ* = 0.36 at 6 months and *ρ* = 0.46 at 12 months; both *p* < 0.001) and were associated with expected nutrient patterns, including higher fiber and monounsaturated fat intake and lower free sugar, saturated fat, and sodium intake. Higher EMA-based adherence was also associated with lower body mass index, visceral adiposity, body fat percentage, insulin resistance, glycated hemoglobin, triglyceride-glucose index, and lifestyle-based dementia risk. The protocol showed high feasibility, with sustained completion rates and positive participant evaluations, although engagement declined modestly over time.

**Conclusion:**

These findings support dietary EMA as a feasible, scalable, and behaviorally meaningful approach for monitoring and promoting Mediterranean diet adherence in real-world lifestyle interventions, with potential value both as an assessment tool and as an active component to reinforce dietary change. The PENSA trial is registered at ClinicalTrials.gov (NCT03978052).

## 1 Introduction

The Mediterranean Diet (MedDiet) is widely recognized as one of the healthiest dietary patterns. Higher adherence to the MedDiet has been associated with a lower risk of cardiovascular diseases (1), neurodegenerative disorders (2), diabetes (3), and cancer (4). Promoting adherence to the MedDiet has therefore become a central component of preventive lifestyle interventions. However, despite its well-established health benefits, sustaining long-term adherence remains a major challenge, highlighting the need for personalized and innovative strategies that effectively support lasting dietary changes (5).

Obtaining accurate dietary data is crucial for understanding the role of nutrition in human health and disease progression (6). However, dietary intake assessment remains inherently complex and affected to various biases. Traditional dietary assessment methods, such as food diaries, food frequency questionnaires, and 24-h recalls, have been widely used in nutritional epidemiology and clinical research, yet accurately capturing dietary exposure through self-reported measures remains highly challenging. These tools are susceptible to misreporting, social desirability and recall biases, are time-consuming, and often place a significant burden on both participants and researchers (7–9). To address these limitations, there is a growing interest in improving traditional dietary assessment approaches to better balance data accuracy and reliability with participant burden and resource efficiency (9).

In this context, ecological momentary assessments (EMA) have emerged as a promising approach for collecting high-frequency, real-time, longitudinal data on human behavior. Initially developed in the field of psychology, EMAs have increasingly been adapted across various disciplines, including nutrition. By capturing behaviors in real-life settings, EMAs reduces reliance on memory and enhances ecological validity (10). Typically delivered through mobile technology, EMAs also represent a scalable and widely accessible assessment method.

The integration of EMAs into dietary assessment offers several advantages, including improved accuracy and reduced reporting and recall bias. It also alleviates participants and researchers’ burden while enabling continuous monitoring of dietary behaviors by capturing temporal fluctuations, intra-individual variability, and evolving patterns over time. This approach has the potential to mitigate reactivity bias, improve participant compliance, and reduce the likelihood of missing data (7, 8, 11, 12).

Nevertheless, EMAs also present important limitations. They are generally more suitable for monitoring dietary behaviors than for estimating precise nutrient or caloric intake (10). Their validity also relies on participants’ ability to respond accurately and consistently, which may require prior training and ongoing support. Moreover, relatively few nutrition studies have validated EMA-based measures against traditional dietary assessment methods, limiting broader implementation and the generalizability of findings (10). Further research is therefore needed to establish standardized protocols, evaluate measurement accuracy, and determine applicability across diverse populations and settings.

This need may be particularly relevant in older adults at risk of cognitive decline. Nutritional interventions are increasingly being explored as strategies for dementia prevention, yet traditional dietary assessment methods rely heavily on memory and sustained participant engagement, challenges that may be exacerbated in individuals experiencing subjective cognitive difficulties. At the same time, the growing adoption of mobile technologies among older adults has created new opportunities for implementing low-burden digital monitoring strategies in this population. Consequently, EMA-based approaches may be especially valuable for supporting and monitoring long-term dietary behavior change in dementia-prevention interventions (13).

Despite their potential, dietary EMA protocols remain highly heterogeneous, and robust validation studies in older adults and long-term real-world intervention are still scarce. To address this gap, we developed and validated a rotating, low-burden dietary EMA protocol to monitor adherence to the MedDiet during a 12-month multimodal lifestyle intervention. We evaluated its feasibility, convergence with Mediterranean Diet Adherence Screener (MEDAS) and food diaries, and its associations with cardiometabolic and anthropometric outcomes.

Importantly, in this study EMAs were used not only as a research tool to assess dietary compliance, but also as a self-monitoring strategy to enhance participants’ awareness of their eating habits, making them an integral component of the nutritional intervention. In parallel, dietary intake was also assessed using traditional methods to enable comparative analyses. Furthermore, the multimodal design of the PENSA study provided a unique real-world framework in which to evaluate dietary EMA within a broader intervention targeting multiple lifestyle behaviors associated with cognitive health.

The primary aim of this report was to validate an EMA-based methodology for monitoring adherence to the MedDiet within the 1-year PENSA multimodal lifestyle clinical trial (14). PENSA combined dietary counseling, physical activity, cognitive training, and social engagement in older adults at increased risk of cognitive decline. A secondary objective was to evaluate its feasibility, performance, and convergence with conventional dietary assessment methods, thereby contextualizing its utility as a monitoring tool. To further contextualize EMA-derived indicators, dietary changes over the intervention were also characterized using food diaries, which served as the quantitative comparator for food groups and nutrient intake.

## 2 Materials and methods

### 2.1 Study design and participants

The PENSA study, a 12-month randomized, double-blind, placebo-controlled clinical trial, served as the platform for the present validation study. Within this trial, all participants received a standardized multimodal lifestyle intervention (diet, physical activity, cognitive training, and social engagement) and were randomized to receive either epigallocatechin gallate (EGCG) supplementation or placebo (14, 15). The trial is part of the global dementia prevention research consortium known as WW-FINGERS (16).

**Figure 1:**
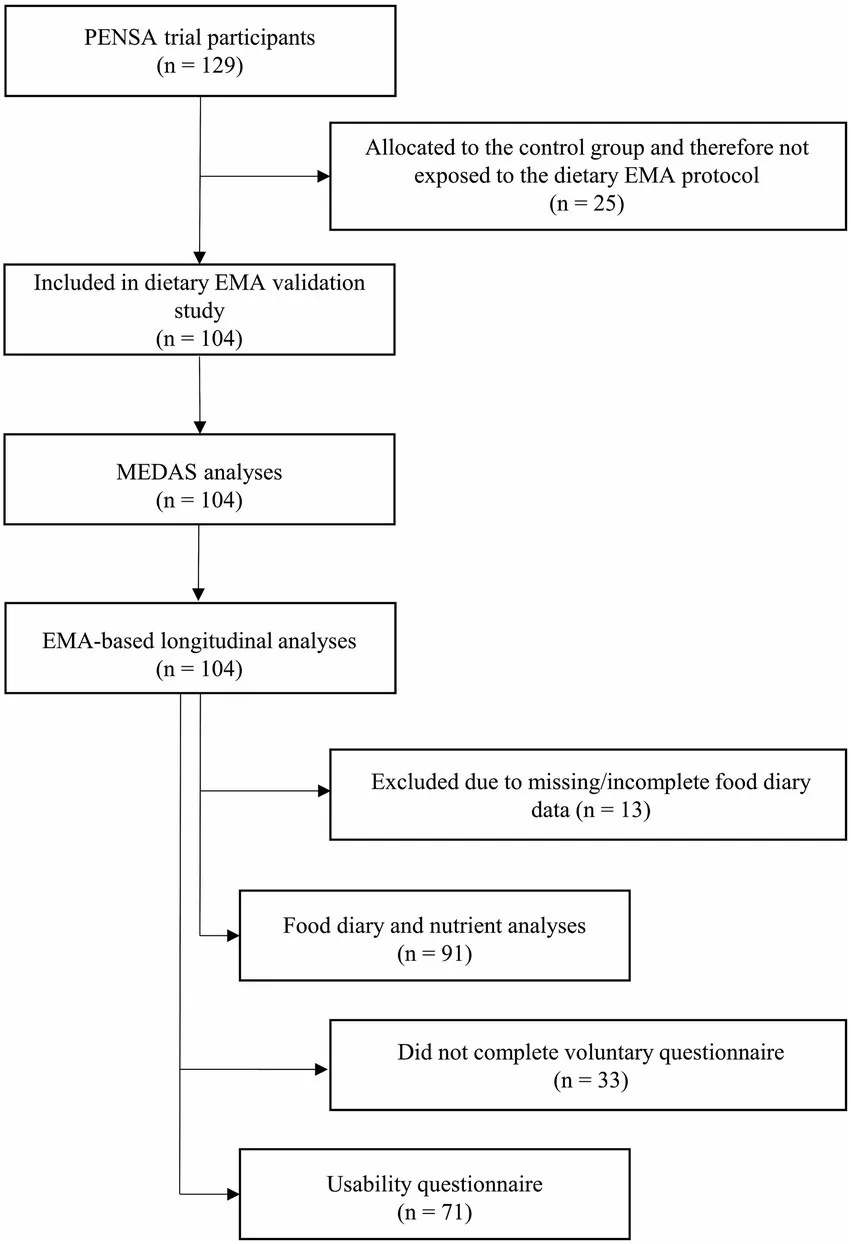
Participant flow diagram for the dietary EMA validation study. Flow diagram showing participant inclusion, exclusions, and sample sizes available for each analysis, including EMA-based longitudinal analyses, MEDAS analyses, food diary and nutrient analyses, and the usability questionnaire.

Eligible participants were men and women aged 60–80 years with subjective cognitive decline (SCD) and confirmed APOE-ε4 carrier status. Exclusion criteria included a history of neurological or psychiatric disorders, active or recurrent malignancies treated within the previous 2 years, medical conditions or medications potentially interfering with study assessments, and a body mass index (BMI) <18.5 or >35 kg/m^2^, among other factors (14). A total of 129 participants were included in the PENSA trial. Of these, 104 participants who completed the multimodal lifestyle intervention and had valid dietary data were included in the present analyses. Participant flow, exclusions, and sample sizes for each analysis are summarized in Figure 1.

The study protocol (2018/8179) was approved by the local institutional review board (CEIm-PSMAR) and conducted in accordance with the ethical principles of the Declaration of Helsinki. The study also complied with European Union regulations for clinical trials and Good Clinical Practice (Directives 2001/20/EC and 2005/28/EC), as well as the General Data Protection Regulation (GDPR, EU 2016/679). All participants provided written informed consent prior to enrolment. The trial is registered at ClinicalTrials.gov (NCT03978052), and the safety of all interventions was closely monitored throughout the study period.

### 2.2 Nutritional intervention overview

The nutritional intervention represented one of the pillars of the multimodal lifestyle intervention program. Each participant received a personalized dietary plan based on the principles of the MedDiet, tailored to their habitual intake patterns, nutritional status, comorbid conditions, and ongoing pharmacological treatments. Dietary counseling was delivered across nine individual 1-h sessions conducted both on-site and online throughout the 12-month intervention period. These sessions aimed to strengthen core dietary goals, support behavioral changes, and adjust recommendations as needed according to participant progress.

**Figure 2:**
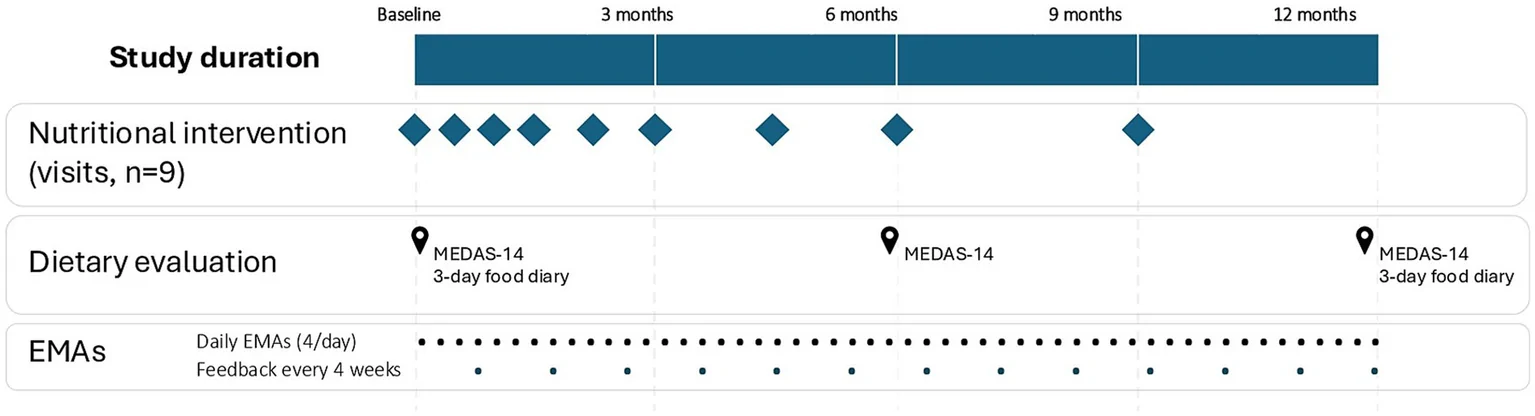
Study timeline and dietary assessment schedule. The intervention lasted 12 months. Nutritional intervention was delivered through individual diet counseling sessions distributed across follow-up (diamonds). Traditional dietary assessment was performed at predefined visits (pin icons): Medas-14 at baseline, 6 and 12 months, and 3-day food diaries at baseline and 12 months. Dietary ecological momentary assessments (EMAs) were collected continuously during follow-up (black dots; evaluation), and periodic personalized feedback written reports were delivered at regular intervals aligned with follow-up cycles (blue dots; feedback, every 4 weeks).

Dietary adherence was assessed using traditional dietary assessment tools at baseline and at multiple timepoints of the intervention, including: (a) the 14-item MedDiet Adherence Screener (MEDAS-14) (17); and (b) a 3-day food diary. In parallel, a novel digital EMAs strategy (c) was implemented to monitor adherence in real time. The design and implementation of this component are described in detail in Section 3, and study timeline is outlined in Figure 2.

Beyond dietary intake, key anthropometric, lifestyle, and cardiometabolic parameters, including BMI, smoking status, alcohol intake, body composition (e.g., visceral fat index and body fat percentage), blood pressure, lipid profile, and markers of glycemic control (fasting glucose and HbA1c)-were assessed using standardized procedures at baseline, 6 and 12 months. Derived scores included insulin resistance estimated using the homeostatic model assessment (HOMA-IR) and the triglyceride-glucose (TyG) index. Dementia risk was evaluated using the LIBRA (LIfestyle for BRAin health) score, a validated composite index based on modifiable risk factor (18). BMI categories were defined according to WHO criteria. These measures provided a broader assessment of the intervention’s impact on overall cardiometabolic health.

### 2.3 EMAs rationale development and implementation

To enable high-frequency, ecologically valid monitoring of dietary adherence throughout the intervention period, a digital EMA protocol was implemented to all participants undergoing the multimodal intervention. Each EMA prompt consisted of four multiple-choice questions addressing participant’s dietary intake over the preceding 24–48 h. In total, eight unique questions were developed and organized into daily sets of four, delivered according to a fixed rotating schedule repeated every 4 weeks. This design ensured comprehensive assessment of eleven food groups within each weekly cycle while minimizing participant burden. To reduce response anticipation, participants were not informed of the rotation pattern. A preliminary description on how EMAs were developed has been described previously (19).

The EMAs assessed intake patterns across eleven predefined food groups central to the MedDiet, including health-promoting components (fruits, vegetables, extra virgin olive oil, whole grains, legumes, nuts, and fish/seafood) and items considered detrimental to health when consumed excessively (sugary drinks, sweets and pastries, and red and processed meats). The scoring framework was based on validated MedDiet adherence metrics, as detailed in Supplementary Table 1 (20). Importantly, the EMA framework was aligned with MedDiet recommendations by capturing both daily and weekly intake frequencies. Food groups recommended for daily intake—such as fruits, vegetables, and extra virgin olive oil—were assessed multiple times throughout the week to capture short-term variability and ensure consistent adherence. For food groups typically consumed on a weekly basis—such as whole grains, sugary beverages, nuts, legumes, sweets and pastries, fish/seafood, and red and processed meats—intake frequency was estimated through repeated sampling distributed across the week. As each EMA prompt referred to the previous 24–48 h, overlapping recall windows enabled reconstruction of weekly dietary patterns without requiring participants to report of all items.

**Figure 3:**
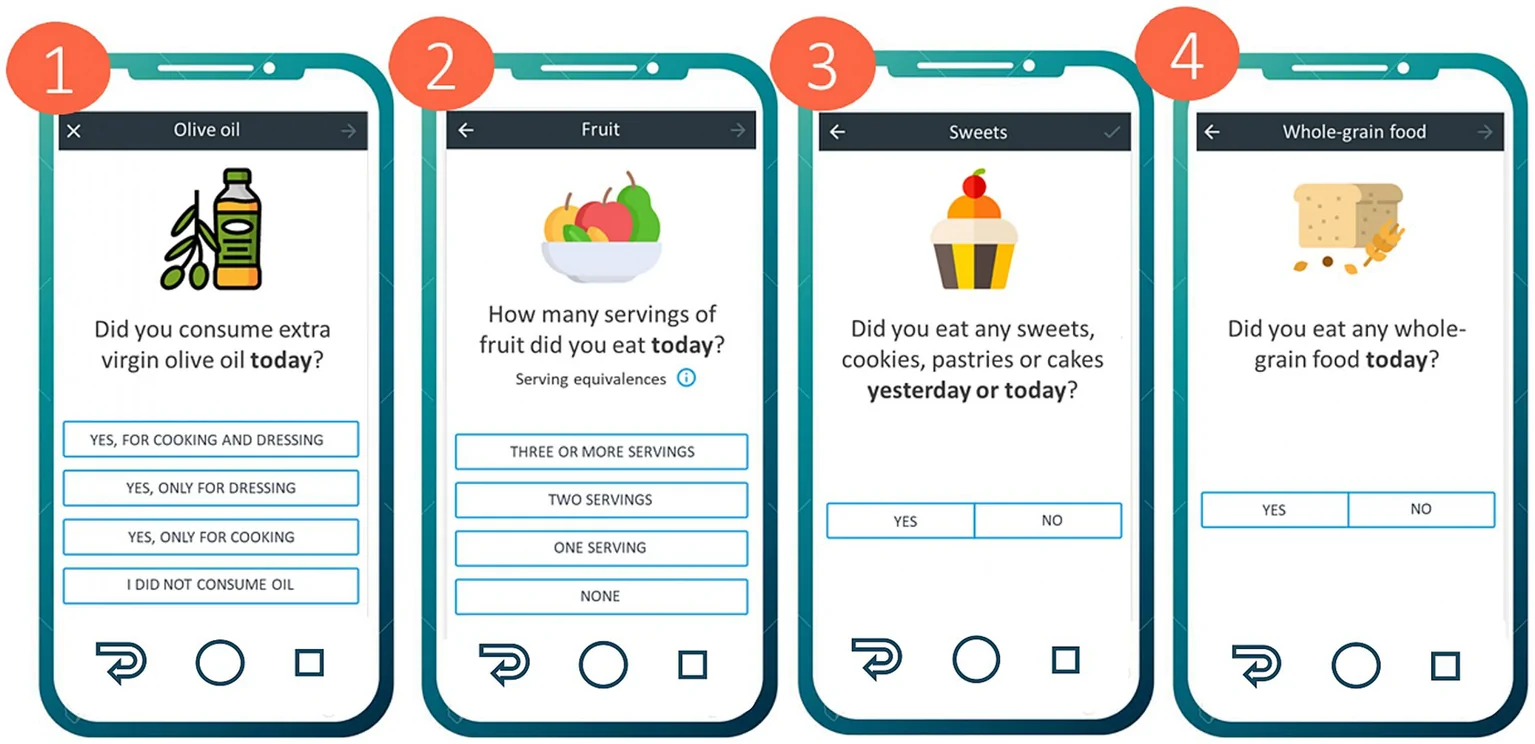
Representative screenshots of the dietary EMA interface. Example items delivered via an SMS link and completed on a smartphone using LimeSurvey, illustrating the main response formats used in the protocol. EMA items rotated according to the predefined schedule described in the Methods.

Every day, participants received EMA prompts at a fixed time via SMS, each containing a personalized link to the LimeSurvey platform,^1^ where questionnaires were completed and responses securely stored. Representative screenshots of the EMA items are shown in Figure 3. Prior to data collection, participants attended a structured training session covering food classification, portion size estimation, and technical instructions for accessing and completing the EMAs (Supplementary Table 2).

Weekly adherence scores to the MedDiet were computed based on EMA responses, with a maximum of 11 points per week. Adherence was categorized as low (≤5), moderate (>5 and <8), or high (≥8). Importantly, once an EMA questionnaire was opened, participants were required to complete all items (no missing responses were allowed). Therefore, adherence was calculated based on the number of days with completed EMAs relative to the total number of prompted days (7 per week), with ≥80% (i.e., ≥6 days/week) considered acceptable. Every 4 weeks, participants received personalized feedback via email, including their 4-week MedDiet adherence score (based on data assessed via EMAs), dietary recommendations for improvement in cases of suboptimal adherence, and a set of seasonal recipes to support dietary goals. This combination of real-time monitoring and feedback was intended to enhance engagement and nutritional awareness, reinforce learning, and promote sustained behavioral changes over the course of the intervention.

### 2.4 Traditional dietary assessment

Conventional dietary assessment methods were used to evaluate dietary intake and food patterns. Three-day food diaries were used as a short-term reference method to quantify energy, nutrients, and food-group intake and to evaluate convergent validity. Participants were instructed to record all foods and beverages immediately after consumption, detailing product types, estimated quantities, and relevant preparation notes. Each diary was subsequently reviewed in detail with a nutritionist during in-person visits to clarify entries and retrieve missing information.

All dietary data were accurately converted into household measures and analyzed using Nutritics® Research Edition v6.01 (Nutritics Ltd., Dublin, Ireland) for nutrient analysis. Mixed dishes were systematically deconstructed into individual ingredients based on standardized household recipes. The resulting data were used to estimate total nutritional intake and servings across predefined food groups.

In addition, the Mediterranean Diet Adherence Screener (MEDAS-14) (17) was administered at baseline, 6, and 12 months through structured interviews conducted by a trained nutritionist to ensure accuracy and consistency.

Although MedDiet adherence indices can also be derived from food records, in this study the 3-day food diaries were primarily collected as a short-term quantitative reference for nutrient and food-group intake rather than for estimating habitual dietary pattern adherence. Given their limited temporal coverage (3 days), food diaries may under-capture infrequently consumed foods that contribute to weekly MedDiet scoring, potentially leading to unstable adherence classification. Therefore, the MEDAS-14 was used as the primary benchmark for dietary adherence, whereas 3-day food diaries were reserved for quantitative comparisons of intake.

### 2.5 Comparative performance analysis

Comparative analyses were conducted to contextualize EMA-derived estimates against established dietary assessment methods within the constraints of the intervention design. As no single dietary assessment method can be considered a universal gold standard, comparator instruments were selected according to the dietary dimension and timeframe of interest.

The MEDAS-14 questionnaire was used as the reference for dietary pattern adherence, as it is a validated instrument in Spanish populations and captures habitual, pattern-level intake over a weekly timeframe. In contrast, 3-day food diaries were used as the reference for quantitative nutrient and food-group intake, as they provide detailed information on portion sizes, preparation methods, and food composition, despite reflecting short-term intake.

Accordingly, EMA performance was evaluated across two complementary domains: (i) convergent validity, referring to the association between EMA-derived indicators and comparator measures, and (ii) agreement, referring to absolute concordance and classification performance.

### 2.6 Feasibility and usability evaluation

The usability of the dietary assessment system was evaluated after 9 months of continuous use. Participants completed an anonymous questionnaire assessing their experience with both the daily EMA-based monitoring and the 4-week feedback reports. The questionnaire was developed based on the Unified Theory of Acceptance and Use of Technology (UTAUT) framework, focusing on key constructs such as perceived usefulness, ease of use, and burden (21). Participants rated aspects of the daily assessments, including clarity of questions, time and effort required, enjoyment, and the tool’s impact on dietary awareness and food choices. Self-reported adherence and perceived barriers to daily completion (e.g., lack of time, motivation, or technical issues) were also documented. For the monthly feedback reports, participants evaluated usefulness, clarity, accuracy, motivational impact, and the appropriateness of delivery frequency.

### 2.7 Statistical analysis

Descriptive statistics were used to summarize baseline characteristics of the study population, including age, sex, and clinical measures. Categorical variables were presented as absolute and relative frequencies (%), while continuous variables were expressed as means with standard deviations (SD). For dietary intake variables, means and 95% confidence intervals (95% CI) were also calculated at baseline and 12 months.

Changes in dietary intake and MedDiet adherence over time were evaluated using linear mixed-effects models. These models accounted for repeated measures within individuals and included fixed effects for time and potential confounders such as age, sex, and intervention group. For subgroup analyses by BMI category, an interaction term between time and BMI category was included in the linear mixed-effects models, using the normal-weight group as the reference category. When overall time effects were significant, pairwise *post hoc* comparisons between time points were performed using estimated marginal means derived from the linear mixed-effects models, with *p*-values adjusted for multiple testing using Tukey’s method. For specific longitudinal analyses, piecewise linear mixed-effects models were additionally fitted to characterize changes across predefined time segments. Effect sizes were reported using Cohen’s d to provide a standardized measure of the magnitude of changes over time, complementing statistical significance.

To explore different domains of EMA performance, we evaluated both convergent validity and agreement with comparator methods. Convergent validity was assessed using Spearman correlations and nutrient/food-group gradient analyses (e.g., diary-derived intakes across EMA score categories). For food-group gradient analyses, diary-derived food intake was examined across ordinal EMA score categories using linear trend tests, with EMA categories treated as an ordered variable, whereas agreement was assessed using classification metrics (sensitivity, specificity, and percent agreement) for dichotomized food-group targets.

Convergent validity between EMA-derived indicators and 3-day food diaries was examined at 12 months. No baseline EMA data were available, as EMA was part of the intervention and responses collected from month 1 onward already reflected early effects of the intervention. Paired *t*-tests were applied to compare mean servings of food groups and EMA-derived adherence indicators with corresponding diary-derived food-group intakes. Agreement was further evaluated using Bland–Altman plots.

Associations between MedDiet EMA total score and clinical/metabolic outcomes at 12 months were evaluated using linear regression models adjusted for age, sex, and treatment allocation. Regression coefficients (*β*) were interpreted as the estimated change in each outcome per one-unit increase in MedDiet EMA total score.

All statistical analyses were conducted in R (Version 4.4.1; R Foundation for Statistical Computing, Vienna, Austria), primarily using the nlme package for mixed-effects models, and multcomp and emmeans for *post hoc* comparisons.

The present study represents a secondary analysis embedded within the PENSA clinical trial. Therefore, the sample size was determined by the objectives and power calculation of the parent trial, as described previously (14), and no additional *a priori* sample size calculation was performed specifically for the EMA validation analyses.

## 3 Results

### 3.1 Participant characteristics

Table 1 presents the descriptive statistics of the total sample (*n* = 104). The total sample had a mean age of 67.3 years, ranging from 60 to 80. Women represented the 66.3% of the sample. Participants had an average of 14.6 years of education (SD = 3.7). The BMI was 26.0 kg/m^2^ (SD = 4.3). Based on BMI classification, 0.96% of individuals were underweight, 47.1% had normal weight, 32.7% were overweight, and 19.2% were classified as obese.

**Table 1:** Baseline characteristics of the study cohort.

Characteristic	All participants (*n* = 104)	Women (*n* = 69)	Men (*n* = 35)
Age, years	67.3 (4.6)	67.6 (4.70)	66.7 (4.53)
Sex, *n* (% women)	69 (66.3%)	–	–
Education, years	14.6 (3.7)	14.8 (3.85)	14.3 (3.44)
BMI (kg/m^2^)	26.0 (4.3)	24.8 (4.45)	27.5 (3.68)^*^
BMI category			
Underweight	1 (0.96%)	1 (1.45%)	0 (0.00%)^*^
Normal weight	49 (47.1%)	39 (56.5%)	10 (28.6%)^*^
Overweight	34 (32.7%)	20 (29.0%)	14 (40.0%)^*^
Obese	20 (19.2%)	9 (13.0%)	11 (31.4%)^*^
Waist circumference (cm)	94.7 (12.1)	91.1 (11.0)	102 (10.7)^**^
Visceral fat index	10.8 (4.0)	9.03 (2.82)	14.3 (3.57)^**^
Alcohol intake (g)	7.19 (7.7)	5.47 (5.55)	9.68 (9.52)
Smoking			
Current Smoker	8 (7.7%)	4 (5.80%)	4 (11.4%)
Former smoker	51 (49.0%)	31 (44.9%)	20 (57.1%)
Never smoker	45 (43.2%)	34 (49.3%)	11 (31.4%)
APOE genotype *n* (%)			
ε2/ε4	8 (7.69%)	5 (7.25%)	3 (8.57%)
ε3/ε4	91 (87.5%)	62 (89.9%)	29 (82.9%)
ε4/ε4	5 (4.8%)	2 (2.90%)	3 (8.57%)

Continuous variables are reported as mean (standard deviation). Categorical variables are reported as counts and percentages. Statistical differences between women and men are indicated as follows: ^*^p < 0.05; ^**^p < 0.001.

### 3.2 Changes in dietary intake and MedDiet adherence

#### 3.2.1 Changes in MedDiet index

**Figure 4:**
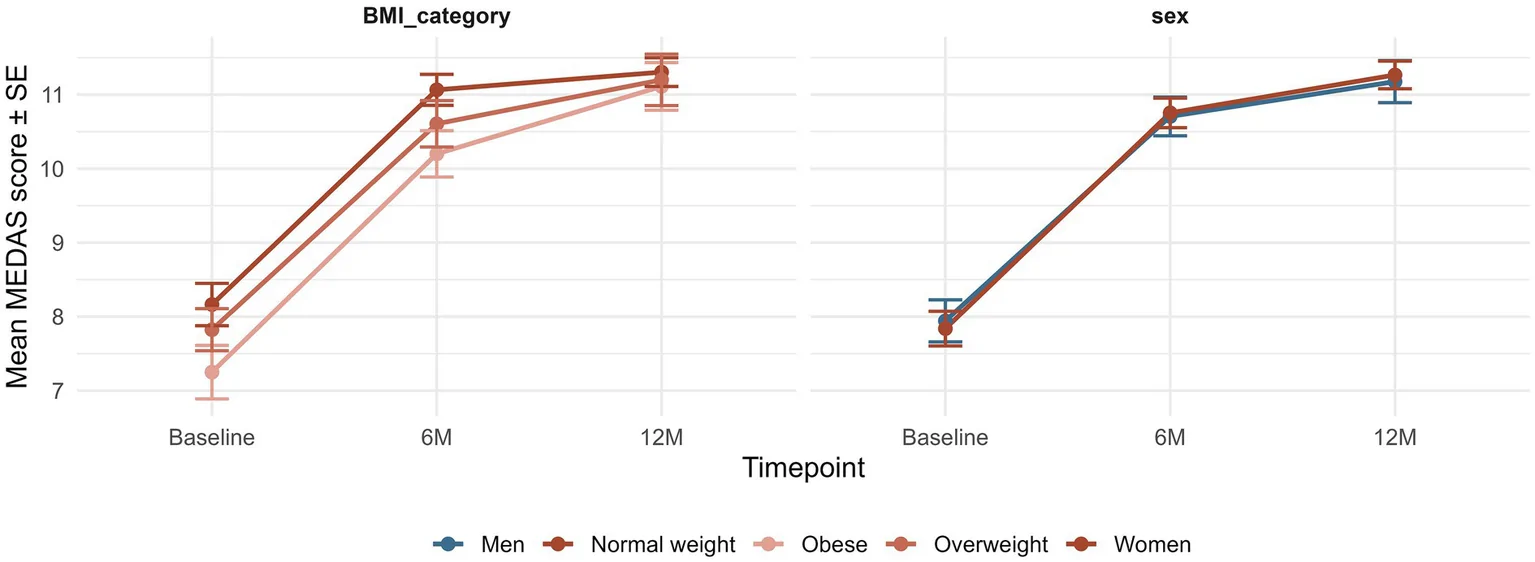
Evolution of MEDAS scores over 12 months **(A)** stratified by BMI category and **(B)** stratified by sex. Values represent mean MEDAS score ± standard error (SE) at baseline, 6 months, and 12 months.

Over time, MEDAS scores rose significantly for the entire sample independently of the sex and BMI categories (Figure 4). Among all participants (*N* = 104), mean MEDAS scores increased from 7.9 (SD = 1.8) at baseline to 10.7 (SD = 1.6) in 6 months and 11.2 (SD = 1.5) at 12 months. Pairwise comparisons between time points, derived from the linear mixed-effects model with Tukey adjustment for multiple testing, showed overall significant increases from baseline to 6 months (*p* < 0.001), from baseline to 12 months (*p* < 0.001), and from 6 to 12 months (*p* = 0.023). Similar improvements were observed when analyzed in both females and males, as well as across different BMI groups. No significant differences in MEDAS scores were found between BMI groups at any time point; however, at baseline there was a trend toward lower MEDAS score in the obese group compared to the normal-weight group (*p* = 0.07).

#### 3.2.2 Changes in the nutrient intake

Food diary–derived changes in nutrient and food-group intake are presented to characterize the dietary shift achieved during the trial and to provide quantitative benchmarks for the convergent analyses with EMA indicators. Nutrient intake analyses included participants with valid food diary records at baseline and 12 months (*n* = 91). Table 2 presents the changes in dietary intake over the intervention period. Overall, total energy and protein intake remained stable (Cohen’s *d* = −0.08 and −0.03, respectively), while total carbohydrate intake decreased modestly (*d* = −0.30), largely due to a marked reduction in free sugars (*d* = −1.13). Fiber intake, on the other hand, increased substantially (*d* = 0.81). The proportion of energy from total fat rose slightly (*d* = 0.37), accompanied by an improvement in fat quality: intakes of MUFA, PUFA, and omega-3 s increased (*d* = 0.35, 0.72, and 0.47, respectively), whereas SFA and trans fats declined (*d* = −0.61 and −0.54). Micronutrient intake also shifted favorably, with higher levels of folate (*d* = 0.41), potassium (*d* = 0.72), and selenium (*d* = 0.27), and a reduction in sodium (*d* = −0.50). Other nutrients such as cholesterol, alcohol, and vitamin B_12_ showed little to no change. Overall, these findings suggest a clear improvement in dietary quality.

**Table 2:** Dietary intake estimates across the study based on the food diaries (*n* = 91).

Nutrient daily intake	Baseline Mean (95% CI)	12 months Mean (95% CI)	Cohen *d*	*p-*value
Energy (Kcal)	1,685.8 (1,617.5, 1,754.2)	1,658.7 (1,593.4, 1,724.0)	−0.08	0.430
Protein (g)	80.0 (76.3, 83.7)	79.4 (75.3, 83.6)	−0.03	0.743
Carbohydrate (g)	151.8 (143.2, 160.4)	142.1 (134.9, 149.2)	−0.3	**0.005**
Free sugars (g)	25.8 (23.0, 28.7)	12.2 (10.2, 14.2)	−1.13	**<0.001**
Fiber (g)	18.9 (17.6, 20.1)	24.6 (22.9, 26.2)	0.81	**<0.001**
Total fat (% TEI)	41.3 (40.2, 42.3)	43.4 (42.2, 44.5)	0.37	**<0.001**
SFA (% TEI)	12.3 (11.7, 12.8)	10.5 (10.0, 10.9)	−0.61	**<0.001**
MUFA (% TEI)	17.9 (17.2, 18.6)	19.2 (18.3, 20.1)	0.35	**0.001**
PUFA (% TEI)	6.6 (6.2, 7.1)	8.8 (8.2, 9.4)	0.72	**<0.001**
Cholesterol (mg)	236.3 (220.6, 251.9)	229.3 (208.6, 250.1)	−0.07	0.517
Trans fats (g)	0.85 (0.71, 1.00)	0.48 (0.39, 0.56)	−0.54	**<0.001**
Omega 3 (g)	1.4 (1.2, 1.6)	2.1 (1.8, 2.4)	0.47	**<0.001**
Alcohol (g)	15.4 (11.6, 19.2)	15.8 (12.1, 19.6)	0.04	0.799
Folate (μg)	227.3 (211.0, 243.6)	261.5 (242.4, 280.5)	0.41	**<0.001**
Vitamin B_12_ (μg)	6.3 (5.7, 6.9)	6.1 (5.2, 7.1)	−0.03	0.769
Sodium (mg)	1,763.4 (1,658.8, 1,867.9)	1,442.9 (1,329.2, 1,556.6)	−0.5	**<0.001**
Potassium (mg)	2,659.3 (2,518.3, 2,800.2)	3,114.5 (2,942.9, 3,286.2)	0.72	**<0.001**
Selenium (mg)	60.1 (55.8, 64.5)	67.7 (61.5, 73.8)	0.27	**0.011**

Results are shown as mean (95% CI). TEI, Total Energy Intake; SFA, Saturated Fatty Acids; MUFA, Monounsaturated Fatty Acids; PUFA, Polyunsaturated Fatty Acid.

Bold values indicate statistically significant differences (*p* < 0.05).

#### 3.2.3 Changes in food group consumption

Changes in the average daily intake of selected food groups from baseline to 12 months are presented in Table 3, as derived from food diary data. Significant increases were observed in the consumption of fruits, vegetables, legumes, nuts, healthy fats, eggs, fish and seafood, and wholegrains. Notably, fruit and vegetable intake increased substantially, with large effect sizes (Cohen’s *d* = 0.87 and 0.82, respectively). Intake of legumes and nuts also rose significantly, alongside a moderate increase in healthy fats, reflecting an overall shift toward more plant-based and nutrient-dense foods. Conversely, consumption of refined cereals, ultra-processed foods, sugar-sweetened beverages, processed meats, and red meat decreased markedly over the study period. The most pronounced reductions were seen in refined cereals (Cohen’s *d* = −0.93), ultra-processed foods (−0.82), and processed meats (−0.74). Dairy and white meat intake remained relatively stable, with no statistically significant changes.

**Table 3:** Average daily intake of food groups at baseline and 12 months based on food diary data (*n* = 91).

Food group daily average intake	Baseline Mean (95% CI)	12 months Mean (95% CI)	Cohen *d*	*p-*value
Fruit	253.3 (218.7, 287.9)	385.3 (347.5, 423.1)	0.87	**<0.001**
Vegetables	222.5 (204.3, 240.7)	300.7 (274.9, 326.5)	0.82	**<0.001**
Legumes	25.6 (20.4, 30.8)	48.0 (38.4, 57.6)	0.45	**<0.001**
Nuts	10.93 (8.8, 13.1)	26.93 (20.7, 33.1)	0.55	**<0.001**
Healthy fat	20.7 (17.9, 22.2)	25.0 (22.8, 27.2)	0.51	**<0.001**
Dairy	184.6 (157.2, 212.0)	171.5 (145.3, 197.7)	−0.17	0.106
Eggs	22.6 (18.8, 26.5)	29.1 (23.2, 35.1)	0.21	**0.044**
Fish and seafood	64.1 (55.8, 72.4)	84.8 (73.4, 96.2)	0.36	**0.001**
White meat	31.3 (24.4, 38.3)	30.3 (22.3, 38.2)	−0.02	0.816
Red meat	31.5 (25.3, 37.7)	21.3 (14.7, 27.8)	−0.32	**0.003**
Processed meat	33.2 (27.7, 38.7)	13.4 (9.0, 17.8)	−0.74	**<0.001**
Refined cereals	108.1 (96.3, 119.9)	56.4 (46.1, 66.8)	−0.93	**<0.001**
Wholegrain cereals	23.1 (17.3, 28.9)	56.3 (47.2, 65.4)	0.73	**<0.001**
Ultra processed food	74.7 (63.2, 86.2)	31.7 (24.9, 38.6)	−0.82	**<0.001**
Sugar sweetened beverages	54.6 (33.7, 75.5)	5.3 (0.14, 10.5)	−0.51	**<0.001**

All results are shown as an average daily intake in grams and 95% CI.

Bold values indicate statistically significant differences (*p* < 0.05).

#### 3.2.4 Changes in EMAs MedDiet score

##### 3.2.4.1 Overall temporal trends

**Figure 5:**
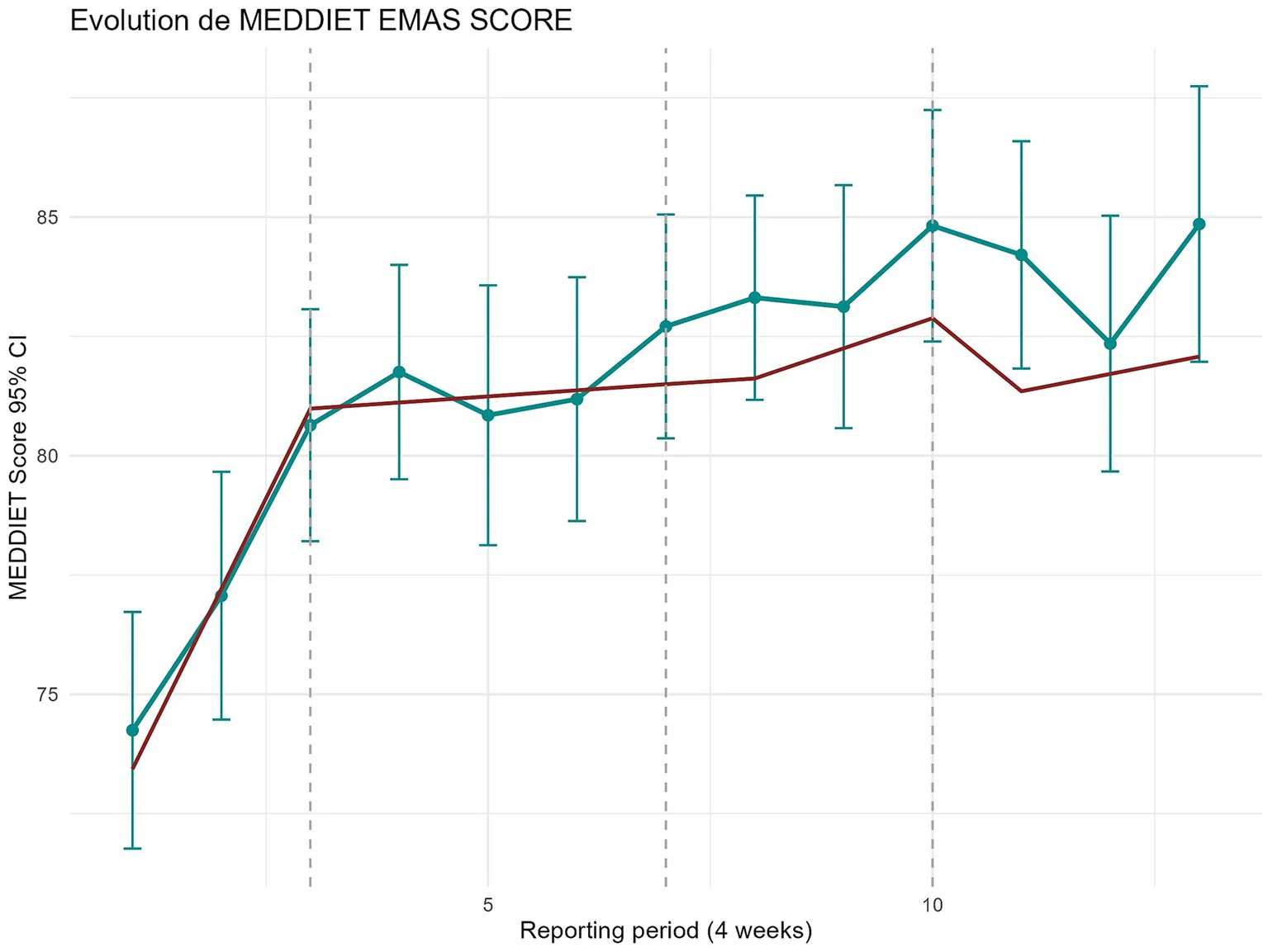
Longitudinal evolution of the MedDiet EMAs score (%) over the 13 4-week reporting cycles. Blue points and lines represent the observed mean scores with their 95% confidence intervals. The red segmented regression line depicts the fitted piecewise linear mixed-effects model with four distinct phases, separated by dashed vertical lines at months 3, 7, and 10, which correspond to the identified breakpoints in the trend.

Figure 5 illustrates the temporal evolution of EMAs-derived MedDiet score over the 12-month period, distributed in thirteen 4-week evaluations, aligning with the feedback periodicity in which it was sent to participants. During the first month of intervention, the EMA-based MedDiet score averaged 73.6% (SD = 11.3), providing the earliest available measure of dietary adherence. The dietary quality score showed a gradual increase across the intervention, reaching an average of 82.3% (SD = 12.5) by the end of the study. To characterize the pattern of change, a piecewise linear mixed-effects model was fitted, dividing the 12-month period into four distinct phases. The model results are summarized in Table 4. The analysis revealed an initial rapid increase (Phase 1), followed by a plateau (Phase 2), a secondary increase (Phase 3), and subsequent stabilization (Phase 4).

**Table 4:** Results of the piecewise linear mixed-effects model showing monthly changes in MedDiet EMAs score (%) across four defined phases.

Phase	Reporting cycle	Estimate (*β*)	Std. error (SE)	*p*-value	Interpretation
Phase 1	1–3	3.78	0.40	<0.001	Strong and significant increase
Phase 2	4–7	0.13	0.19	0.501	No significant change (plateau)
Phase 3	8–10	0.63	0.26	0.016	Moderate significant increase
Phase 4	11–13	0.36	0.26	0.168	No significant change (plateau)

##### 3.2.4.2 Completion rates

The completion rate for EMA dietary questions showed a clear temporal pattern over the course of the study. In the initial months, response rates were high, with an average of 93.2% (SD = 8.6) at Month 1, gradually declining over time to 86.6% (SD = 15.9) by the end of the intervention. Linear mixed-effects modeling confirmed a significant monthly decrease (*β* = −0.51, SE = 0.07, *p* < 0.001), corresponding to an average reduction of ~0.5 percentage points per month, indicating a progressive decline in participant engagement over time. Supplementary Figure 1 shows completion rates across sex and BMI categories, with no clear differences observed between groups.

##### 3.2.4.3 Differences by BMI category

The longitudinal analysis, using “Normal weight” as the reference category, showed MedDiet EMA score improved over time among participants with normal weight (*β* = 0.58, *p* < 0.001). However, participants with obesity exhibited a slower rate of improvement over time compared to those with normal weight (interaction *β* = −0.33, *p* = 0.023). No significant differences in the rate of change were observed for participants classified as overweight. Baseline MedDiet scores did not differ significantly between BMI categories.

At 12 months, model-based pairwise comparisons showed that participants with obesity had lower MedDiet EMA scores than those with normal weight (mean difference: 8.0 points, *p* = 0.046). Scores for overweight participants did not differ significantly from those of the normal-weight group. There was also a tendency for higher scores in the overweight group compared with the obese group (difference: 8.2 points, *p* = 0.060). Descriptively, the observed mean (SD) MedDiet EMA scores at 12 months were 75.1 (2.8) in participants with obesity, 83.3 (2.2) in those with overweight, and 83.0 (1.8) in those with normal weight.

### 3.3 Convergent validity (EMA vs. 3-day food diaries)

#### 3.3.1 EMA vs. MEDAS scores

At baseline, the correlation between the EMA-based MedDiet score and the MEDAS score was weak and not statistically significant (rho = 0.10, *p* = 0.31), likely reflecting that participants had already begun dietary changes during the first month of intervention. By 6 months, the association strengthened to a moderate positive correlation (rho = 0.36, *p* < 0.001), and by 12 months, it further increased to a moderate-to-strong correlation (rho = 0.46, *p* < 0.001), indicating improved agreement between the two dietary assessment methods over time.

#### 3.3.2 Agreement between EMA and MEDAS (Bland–Altman)

**Figure 6:**
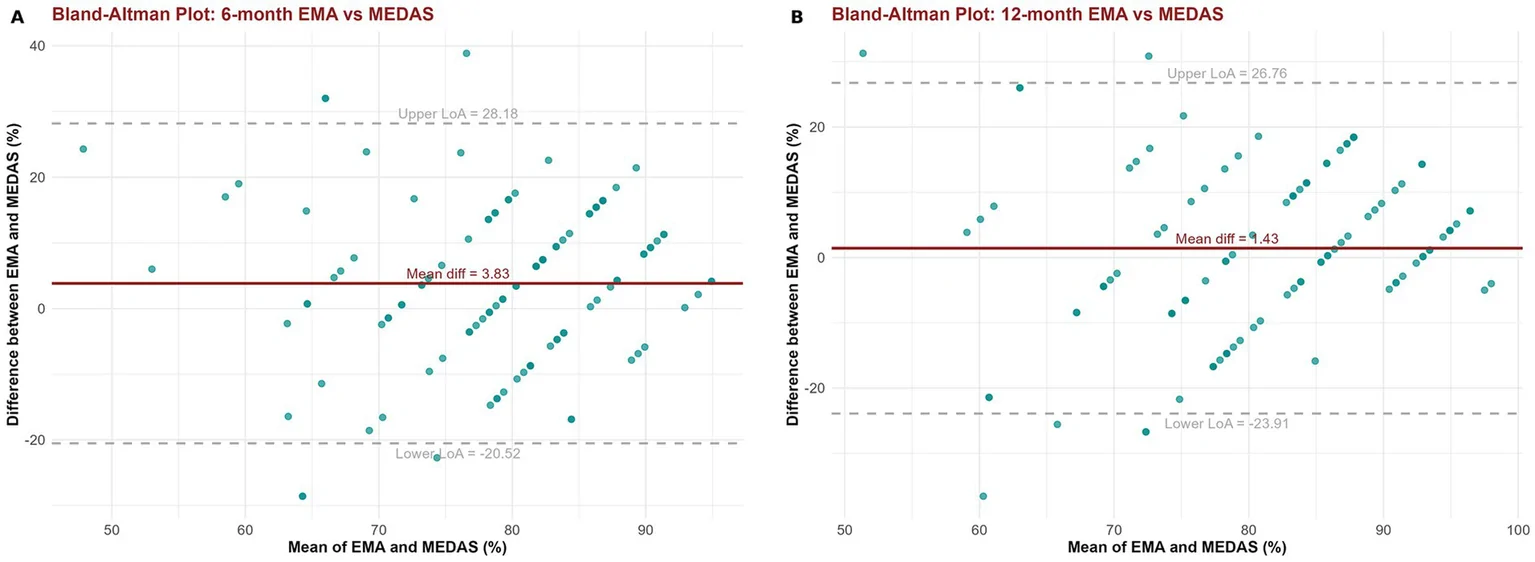
Bland–Altman plots comparing diet quality adherence measured by EMA and MEDAS at 6 **(A)** and 12 months **(B)**.

At 6 months, Bland–Altman analysis comparing MedDiet EMA and MEDAS scores (expressed as percentage) showed a mean difference (bias) of 3.8% (Figure 6A). The standard deviation of the differences was 12.4%, resulting in 95% limits of agreement (LoA) from −20.5 to +28.2%. At 12 months, the bias decreased to 1.4% (Figure 6B), with a standard deviation of 12.9 and 95% LoA ranging from −23.9 to +26.8%.

#### 3.3.3 EMA scores and nutrient profiles

At 12 months, the EMA-derived MedDiet score showed positive associations with fiber (*ρ* = 0.19, *p* = 0.005), MUFA (*ρ* = 0.18, *p* = 0.008), and folate intake (*ρ* = 0.14, *p* = 0.038; Supplementary Table 3). In contrast, inverse correlations were observed with free sugar (*ρ* = −0.24, *p* < 0.001), SFA (*ρ* = −0.18, *p* = 0.008), and sodium intake (*ρ* = −0.18, *p* = 0.009). No significant associations were found with total energy, protein, or most other micronutrients. Overall, these patterns align with the expected nutrient profile of a Mediterranean dietary pattern.

#### 3.3.4 EMA scores and food group intakes

**Figure 7:**
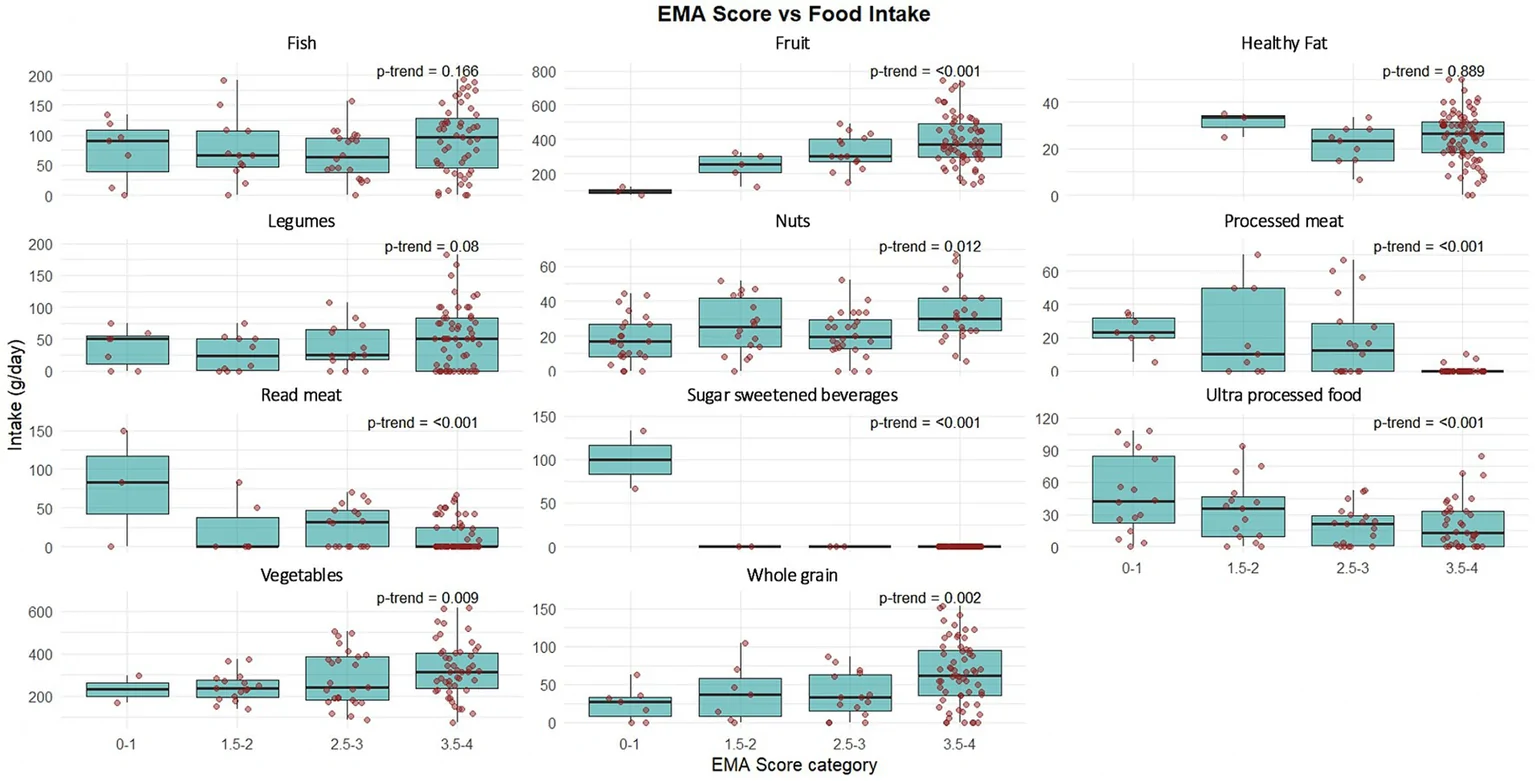
Comparison between EMA scores and food intake across food groups. Boxplots show the distribution of average daily intake (g/day) for each food group according to EMA score categories (0–1, 1.5–2, 2.5–3, 3.5–4). Data on food intake were extracted from the 3-day food diary at 12 months.

Finally, EMA-derived MedDiet scores for individual food categories (ranging from 0 to 4) were compared with average intakes (g/day) reported in the 3-day food diaries. Because EMA scores and diary-derived food intakes were obtained using independent assessment methods, these analyses were intended to examine convergent validity by assessing whether diary-based intake varied in the expected direction across EMA score categories. Significant positive trends were observed for fruit, vegetables, wholegrains, and nuts. In contrast, significant negative trends were found for categories in which higher EMA scores reflected lower consumption, such as sugar-sweetened beverages, sweets and pastries, and red and processed meat. No significant trends were observed for extra-virgin olive oil, fish, or legumes (Figure 7).

#### 3.3.5 Agreement analysis: EMA scores vs. 3-day food diaries

The agreement between EMA scores and 3-day food diaries varied across food groups (Table 5) at 12 months. For foods encouraged in the MedDiet (vegetables, fruit, wholegrains, legumes, and fish), EMAs demonstrated high sensitivity (0.79–0.94) but lower specificity, reflecting the small number of participants with low intake. Nuts showed moderate agreement (58.2%). For limiting foods, agreement was generally lower. Sugar-sweetened beverages and sweets and pastries had poor discrimination, whereas red and processed meat exhibited modest agreement, with specificity higher than sensitivity, indicating EMAs were more effective at identifying non-consumers than low consumers.

**Table 5:** Agreement between EMA scores and 3-day food diary intakes for selected food groups at 12 months.

EMA vs 3-day diary: agreements by food category											
Food	Direction	Cutoff		Performance			Counts				
		EMA	Diary	% Agree	Sens.	Spec.	TP	FP	TN	FN	N
EVOO	High EMA = high intake	3	15	81.3	0.95	0.07	73	13	1	4	91
Vegetables	High EMA = high intake	3	300	61.5	0.82	0.45	33	28	23	7	91
Fruit	High EMA = high intake	3	300	71.4	0.92	0.32	55	21	10	5	91
Whole-grain cereals	High EMA = high intake	3	50	61.5	0.94	0.26	45	32	11	3	91
Sugary drinks (including juices)	High EMA = low intake	3	50	7.70	0.06	0.50	5	2	2	82	91
Sweets and pastries	High EMA = low intake	3	15	39.6	0.46	0.35	17	35	19	20	91
Legumes	High EMA = high intake	3	50	57.1	0.83	0.28	40	31	12	8	91
Nuts	High EMA = high intake	3	10	58.2	0.57	0.65	42	6	11	32	91
Fish and seafood	High EMA = high intake	3	50	60.4	0.79	0.21	49	23	6	13	91
Red meat	High EMA = low intake	3	40	34.1	0.25	0.58	16	11	15	49	91
Processed meat	High EMA = low intake	3	15	33.0	0.30	0.40	18	18	12	43	91

Cutoff refers to the threshold used to classify intake as “high” or “low,” expressed as either EMA score (points) or average daily intake from the 3-day food diaries (grams/day). Agreement (%) reflects the proportion of participants classified similarly by EMA and diaries. Sensitivity denotes the proportion of true high consumers correctly identified by EMA, and specificity the proportion of true low consumers. TP, true positives; FP, false positives; TN, true negatives; FN, false negatives; N, total sample; Sensitivity = TP/(TP + FN) and Specificity = TN/(TN + FP).

### 3.4 Predictive validity: association with the health parameters

Higher scores to MedDiet EMA score at 12 months show expected directional associations supporting construct (predictive) validity of the EMA-derived adherence indicator within the context of the trial (Table 6). Specifically, scores were associated with lower BMI, visceral fat index (fat surrounding internal organs in the abdominal cavity-on a scale from 1 to 59), and body fat percentage, as well as reduced insulin resistance (HOMA-IR), lower HbA1c, and a more favorable triglyceride-glucose (TyG) index. Importantly, adherence was also linked to a lower LIBRA score, reflecting a healthier dementia risk profile.

**Table 6:** Construct validity: associations between MedDiet EMA score and clinical/metabolic outcomes at 12 months.

Outcome	Estimate (*β*)	Std. error	*p*-value
BMI (kg/m^2^)	−0.067	0.032	0.039
Visceral fat index	−0.058	0.022	0.010
Fat percentage (%)	−0.136	0.046	0.004
LIBRA score	−0.080	0.034	0.021
HbA1c (%)	−0.012	0.004	0.005
HOMA-IR	−0.051	0.015	0.001
TyG index	−0.015	0.004	0.001

Linear regression models adjusted for age and sex and treatment allocation (multimodal lifestyle intervention plus EGCG vs multimodal lifestyle intervention plus placebo). *β* coefficients represent the estimated change in each outcome variable, expressed in its original measurement units, associated with a one-point increase in the MedDiet EMA total score. BMI, body mass index; LIBRA, LIfestyle for BRAin health score; HbA1c, glycated hemoglobin; HOMA-IR, homeostatic model assessment of insulin resistance; TyG index, triglyceride-glucose index.

### 3.5 Feasibility and usability of the EMA implementation

**Figure 8:**
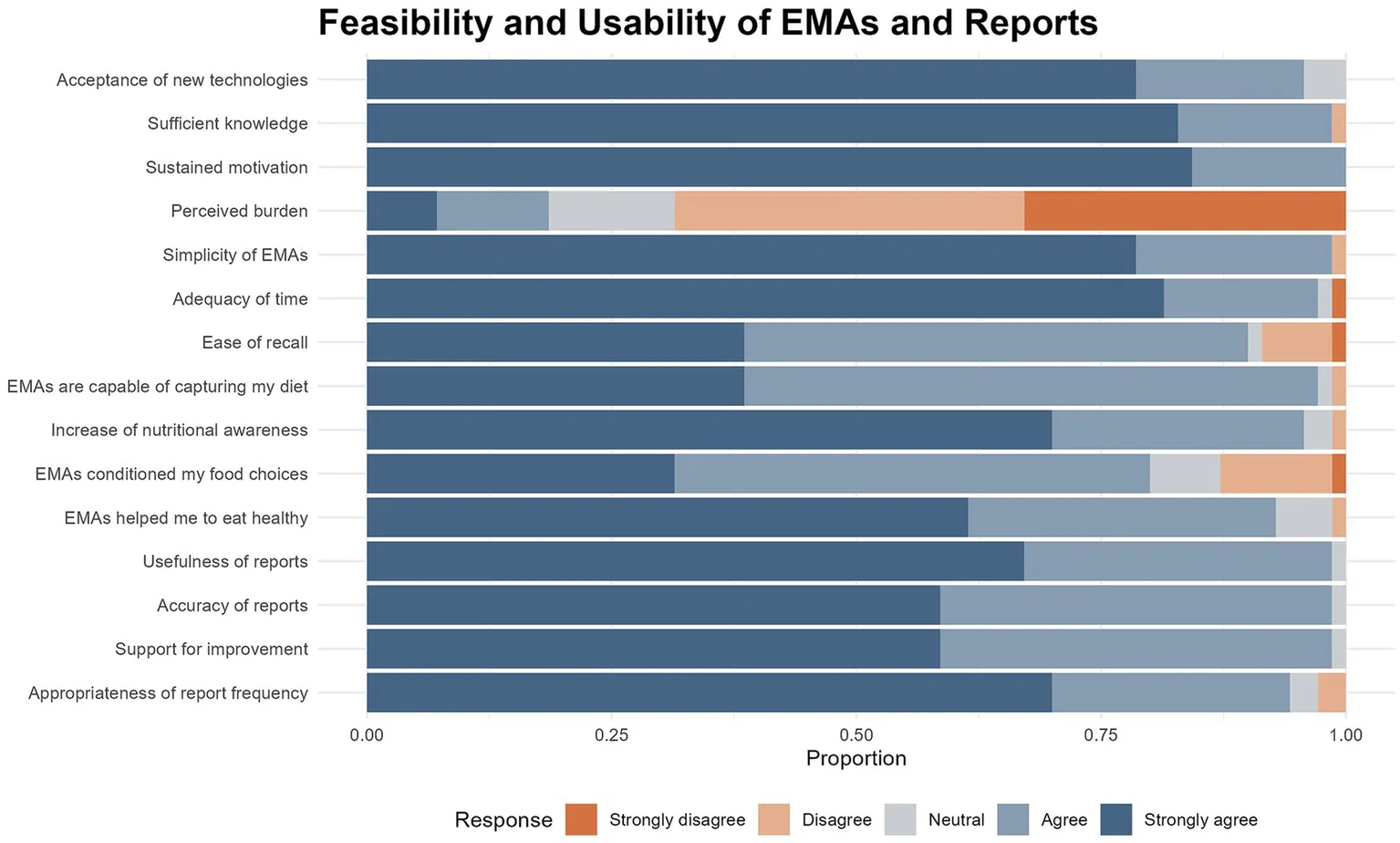
Participants’ evaluations of the feasibility and usability of EMAs and reports after 9 months (*n* = 71). Stacked bars represent the proportion of responses across five Likert categories (“strongly disagree” to “strongly agree”) for each domain assessed.

Participants (*n* = 71) voluntarily and anonymously evaluated the EMAs and reports positively after 9 months of use (Figure 8). Most respondents agreed or strongly agreed that the EMAs were simple to use, that they possessed sufficient knowledge and motivation, and that the time required was appropriate. The EMAs were also perceived as effective in capturing dietary intake, increasing nutritional awareness, and promoting healthier eating habits. Reports were generally rated as useful, accurate, and supportive of improvement. The only area with more varied opinions, was perceived burden: while many did not consider the EMAs demanding, approximately one third of participants reported some degree of burden.

## 4 Discussion

In this 12-month multimodal lifestyle intervention, we developed and implemented a mobile EMA protocol to monitor adherence to key MedDiet food groups, combined with periodic and personalized feedback. Over time, the EMA-based MedDiet adherence score increased rapidly during the first 3 months of the program, plateaued in the middle, and then increased again at the end of the year.

Convergent validity was supported by moderate correlations with MEDAS-14 at 6 and 12 months, as well as by nutrient profiles consistent with a MedDiet pattern (higher fiber and MUFA, lower free sugars and SFA). Moreover, EMA scores at 12 months were associated with favorable anthropometric and metabolic outcomes, including BMI, fat mass, HOMA-IR, HbA1c and TyG index, as well as a lower LIBRA score, reflecting a healthier dementia-risk profile. Together, these findings support the utility of EMA-derived indicators as sensitive, behaviorally grounded markers of dietary adherence in lifestyle interventions.

From a methodological perspective, our findings reinforce the value of EMA for dietary assessment. High-frequency, context-based data collection reduces reliance on long-term memory and enhances ecological validity compared with traditional retrospective instruments. Consistent with prior evidence, EMA reduces recall bias and captures intraindividual variability in real time, although protocols remain highly heterogeneous in terms of prompting schedules and recall windows (8, 12).

Importantly, adherence in our study remained high over 12 months, with a mean completion rate of 86.6%, which is noteworthy given that compliance typically declines over time in long-term EMA protocols. Previous studies have reported progressive attrition, particularly in mid- to long-term monitoring, making sustained adherence a known challenge in EMA-based designs (22, 23).

The accuracy and consistency of dietary self-reporting can vary substantially across food groups and assessment methods. In our study, agreement between EMA-derived dietary scores and traditional assessment tools was domain-specific and varied depending on both the type of food and consumption frequency. Stronger associations were observed for regularly consumed food groups (fruits, vegetables, wholegrains, nuts), whereas weaker discrimination was found for occasionally consumed or socially sensitive items (sugary beverages, sweets/pastries, red/processed meats). These findings align with well-established limitations of dietary self-report methods. Underreporting of foods perceived as unhealthy has been widely reported and is largely attributed to social desirability bias (24).

A key methodological explanation is the mismatch in temporal coverage between diet monitoring instruments. EMAs capture dietary behaviors across all days of the week through repeated 24–48-h window, whereas food diaries represent a short and non-exhaustive snapshot of intake. Even when standardized per day, this narrower window may underrepresent occasionally consumed foods, leading to weaker agreement for low-frequency items. This reinforces that EMA and food diaries capture complementary, rather than interchangeable, dimensions of dietary intake.

The choice of reference methods was therefore driven by the conceptual distinction between short-term intake measures and weekly dietary pattern assessment. MEDAS-14 was used as the benchmark for dietary pattern adherence due to its validation in Spanish populations and its capacity to capture habitual weekly intake. In contrast, 3-day food diaries were used as a quantitative reference for nutrient and food-group intake. Accordingly, MEDAS-14 and food diaries served distinct but complementary roles in evaluating convergent validity and agreement, reflecting different conceptual targets rather than a single unified validity construct.

This work highlights several design features with potential relevance for future dietary interventions. First, rotating EMA prompts aligned with daily versus weekly MedDiet recommendations allowed comprehensive dietary coverage while minimizing participant burden. Second, overlapping 24–48 h recall windows enabled reconstruction of weekly intake patterns without requiring full food logging. Third, periodic personalized feedback supported behavioral reinforcement by providing goals and progress summaries. These components align with evidence that prompts/cues, feedback/monitoring, credible source, personalization and human support are key drivers of effectiveness in digital interventions (25).

An additional advantage of the present EMA framework is its suitability for capturing the MedDiet-specific constructs that are difficult to assess using image-based methods. In particular, the assessment of EVOO as a culinary fat bypasses current limitations of photo or image-based methods, which still struggle to disaggregate ingredients in mixed dishes or accurately identify oil or fat type and quality in free-living conditions (26).

Participants reported high acceptability, rating the system as simple, useful and minimally burdensome, alongside increased nutritional awareness. A small but significant decline in completion rates over time is consistent with patterns observed in long EMA protocols and has been observed in diverse populations. In older adults specifically, pooled EMA datasets show average compliance around 70–85% with diurnal variations and subgroup patterns (e.g., lower evening response), underscoring the need for adaptive engagement strategies to sustain long-term adherence (22). Prior work also indicates that app-based EMA can be feasible and valid in older adults for behaviors such as physical activity, including in noncontact formats—consistent with the high acceptability we observed (22).

This study has several strengths. It was conducted within a 12-month rigorously designed trial, integrating intensive longitudinal monitoring. EMA items were aligned with national dietary guidelines, complemented by structured feedback loops, and validated against multiple reference standards, including MEDAS, food diaries, nutrient profiles, and clinical biomarkers.

Several limitations merit careful consideration. First, it is important to note that the present study was not designed to test the effectiveness or superiority of EMA as a dietary assessment tool over traditional dietary assessment methods. Because effectiveness was not operationally defined and no formal comparator was included, our findings should be interpreted as evidence of feasibility, acceptability, and convergence with traditional methods rather than as proof of enhanced performance over alternative approaches. Second, all dietary tools were self-reported and remain susceptible to reactivity and social desirability bias; while near-real-time EMA likely reduces recall bias, it can still prompt behavior change during assessment. In addition, although EMAs were designed to capture near real-time intake, participants were able to complete questionnaires after the initial prompt, which may have introduced a degree of short-term recall bias. Third, nonrandom missingness is plausible in long protocols (e.g., selective skipping on less healthy days), which could bias weekly scores; sensitivity analyses with inverse probability weighting or multiple imputation are advisable. Fourth, construct coverage was purposely limited to priority MedDiet components; thus, EMA did not capture some domains of interest (e.g., wine/alcohol patterning, dairy, eggs, …), which may contribute meaningfully to health outcomes. Fifth, baseline EMA was unavailable by design, as EMA was itself an intervention component, meaning early measurements already reflect behavior change. Finally, generalizability may be limited to older adults engaged in a structured multimodal intervention, requiring replication in broader community cohorts.

Overall, the use of EMA to monitor dietary adherence represents a promising tool for scalable digital lifestyle interventions. The MedDiet EMA-based scores provide a pragmatic, temporally sensitive indicator of dietary behavior. However, sustained engagement remains a key challenge. Evidence consistently reports modest linear declines in EMA compliance over time, highlighting the need for richer engagement strategies beyond simple timing personalization—such as micro-incentives, brief gamification elements, or just-in-time adaptive interventions that tie feedback to context (27, 28).

To enhance both adherence and data quality, future research should focus on optimizing feedback frequency and format (e.g., monthly vs. bi-weekly), developing adaptive prompting schedules that account for circadian and weekly response patterns, and integrating hybrid models that combine EMA with low-burden passive or image-assisted inputs for specific dietary targets. Additionally, standardized EMA protocols and validation studies in more diverse populations are needed to facilitate broader implementation and comparability across settings.

## 5 Conclusion

A diet-focused EMA approach, embedded within a multimodal lifestyle intervention and combined with periodic feedback, provided a feasible and valid method for longitudinally monitoring MedDiet adherence in older adults at risk of cognitive decline. Beyond feasibility and validity, this approach shifts the emphasis of dietary assessment from precise portion-size and nutrient quantification (domains highly prone to substantial reporting errors) to the evaluation of food-based behaviors and dietary quality.

Importantly, by increasing nutritional awareness in participants through frequent, contextualized prompts and regular personalized feedback, the EMA system likely also enhanced nutritional awareness and supported behavioral changes targeted by the intervention. EMA-derived scores tracked diet quality over time and aligned with improvements in metabolic and dementia-risk profiles, supporting their dual role as both an assessment tool and an active engagement component within lifestyle programs.

Collectively, these findings highlight the potential of EMA-based methods as scalable tools for supporting and monitoring dietary change in real-world interventions.

## Data Availability

The raw data supporting the conclusions of this article will be made available by the authors, without undue reservation.

## Footnotes

1 https://www.limesurvey.org/.
