# Supplementary material for: Dietary ecological momentary assessments: validation against food diaries and associations with health biomarkers
Source: Front Nutr. 2026 Sep 4;13:1927498. doi: 10.3389/fnut.2026.1927498 (PMC13585530; doi:10.3389/fnut.2026.1927498)
Supplement: Supplementary file 1 [file Supplementary_file_1.docx]

SUPPLEMENTARY MATERIAL

**Supplementary Table 1.** Descriptive Overview of EMAs: Food Groups, Enquiry Frequency, Rationale, and Scoring System

| **Nr**  **Question** | **Food group** | **Type of question** | **Interval of enquiry** | **Weekly frequency of the question** | **Proposed with Recommendations** | **Score** | **MedDiet scoring criteria** |
| --- | --- | --- | --- | --- | --- | --- | --- |
| Q_1_ | EVOO | Qualitative  (Yes/No and usage) | Last 24 hours | 4 times | EVOO as the main source of fat in each meal | 1 | ≥ 75 % ^1^ |
|  |  |  |  |  |  | 0.5 | ≥ 50% |
|  |  |  |  |  |  | 0 | < 50% |
| Q_2_ | Vegetables | Semi-Quantitative (Nº of servings) | Last 24 hours | 4 times | 2 servings/day | 1 | ≥ 75 % ^1^ |
|  |  |  |  |  |  | 0.5 | ≥ 50 |
|  |  |  |  |  |  | 0 | < 50% |
| Q_3_ | Fruit | Semi-Quantitative (Nº of servings) | Last 24 hours | 4 times | 3 servings/day | 1 | 75 % ^1^ |
|  |  |  |  |  |  | 0.5 | ≥ 50% |
|  |  |  |  |  |  | 0 | < 50% |
| Q_4_ | Whole-grain food | Qualitative  (Yes/No) | Last 24 hours | 3 times | Preference for whole-grain foods | 1 | ≥ 66 % ^2^ |
|  |  |  |  |  |  | 0 | < 66% |
| Q_5_ | Sugary drinks (including juice) | Semi-Quantitative (Nº of servings) | Last 24 hours | 3 times | Occasional and moderate consumption | 1 | ≥ 66 % ^3^ |
|  |  |  |  |  |  | 0 | < 66% |
| Q_6.1_ | Legumes | Semi-Quantitative (Nº of servings) | Last 48 hours | 3 times | 2-4 servings/week | 1 | ≥ 2 times/week |
|  |  |  |  |  |  | 0 | < 2 times/week |
| Q_6.2_ | Nuts | Semi-Quantitative (Nº of servings) | Last 48 hours | 3 times | ≤3 servings/week | 1 | ≥ 3 times/week |
|  |  |  |  |  |  | 0.5 | ≥ 2 times/week |
|  |  |  |  |  |  | 0 | < 2 times/week |
| Q_7_ | Sweets and pastries | Qualitative  (Yes/No) | Last 48 hours | 3 times | Occasional and moderate consumption | 1 | ≥ 66 % ^3^ |
|  |  |  |  |  |  | 0 | < 66% |
| Q_8.1_ | Fish and seafood | Semi-Quantitative (Nº of servings) | Last 48 hours | 4 times | 2-3 servings/week | 1 | ≥ 2 times/week |
|  |  |  |  |  |  | 0 | < 2 times/week |
| Q_8.2_ | Red meat | Semi-Quantitative (Nº of servings) | Last 48 hours | 4 times | Occasional and moderate consumption | 1 | ≤ 1 time/ week ^4^ |
|  |  |  |  |  |  | 0 | > 2 times/week |
| Q_8.3_ | Processed meat | Semi-Quantitative (Nº of servings) | Last 48 hours | 4 times | Occasional and moderate consumption | 1 | ≤ 1 time/ week ^4^ |
|  |  |  |  |  |  | 0 | > 1 time/ week |

* Recommendations are based on *Dietary guidelines for the Spanish population (*Spanish Society on Community Nutrition*, December 2016)*

^1.^ We considered a good adherence to the recommendation when subjects complied with more than 75% with the recommendations.

^2.^ We considered a good adherence to the recommendations when whole-grain products were consumed in two or more occasions of the 3 days asked

^3.^ We considered that the consumption was occasional when the intake of sugary drinks and sweets/pastries reported was less than once out of the 3 times asked per week.

^4.^ We considered that the consumption was occasional and moderate when the total number of servings reported in the week was ≤1.

**Supplementary table 2.** **Estimation of serving sizes, and technical instructions for accessing and completing the EMAs** (S, serving)

| **No.** | **Question** | **Serving Size (s)** | **Food Included** | **Food Excluded** |
| --- | --- | --- | --- | --- |
| Q1 | Extra virgin olive oil (EVOO) | Not applicable | Extra virgin olive oil | Olive oil |
| Q2 | Vegetables | 1 s = 200g, ½ s = side dish | Salads, cooked vegetables, sofrito, frozen vegetables | Potato, sweet potatoes, peas |
| Q3 | Fruit | 1 s = 1 medium piece, 1 slice of melon/watermelon, 2-3 small pieces | All fruits (raw, cooked) | Juices, yogurt with fruits, jam |
| Q4 | Whole-grain food | Not applicable | All whole grain products | Refined cereals, non-whole grain multigrain products |
| Q5 | Sugary drinks (including juices) | 1 s = 1 glass | Soft drinks (sugar and sugar-free), natural/ packaged juices | Water, alcoholic drinks |
| Q6.1 | Legumes | 1 s = 150g | Dried/cooked legumes, peas, tofu | Corn |
| Q6.2 | Nuts | 1 s = 25g | All nuts | Dried fruit |
| Q7 | Sweets and pastries | Not applicable | Homemade and industrial baked goods | Not applicable |
| Q8.1 | Fish and seafood | 1 s = 100-150g fish, 1 s = 200g seafood | Raw, frozen, canned, smoked fish and seafood | Surimi and derivatives |
| Q8.2 | Red meat | 1 s = 100-150g | Bovine, game, viscera, duck | Chicken, turkey, lean pork cuts |
| Q8.3 | Processed meat | 1 s = 50g | Sausages, ham, mince, cured meat | Not applicable |

**Supplementary table 3:** Association of MedDiet EMAs Score with Nutrient Intake at 12 Months

| **Nutrient daily intake** | **Spearman rho** | **P-value** |
| --- | --- | --- |
| Energy (Kcal) | -0,054 | 0,433 |
| Protein (g) | 0,128 | 0,065 |
| Carbohydrate (g) | -0,134 | 0,053 |
| Free Sugars (g) | -0,241 | **0,000** |
| Fiber (g) | 0,193 | **0,005** |
| Total Fat (% TEI) | 0,096 | 0,167 |
| SFA (% TEI) | -0,183 | **0,008** |
| MUFA (% TEI) | 0,182 | **0,008** |
| PUFA (% TEI) | 0,087 | 0,208 |
| Cholesterol (mg) | 0,033 | 0,637 |
| Trans Fats (g) | -0,116 | 0,094 |
| Omega 3 (g) | 0,091 | 0,187 |
| Alcohol (g) | -0,143 | 0,109 |
| Folate (µg) | 0,143 | **0,038** |
| Vitamin B_12_ (µg) | 0,110 | 0,112 |
| Sodium (mg) | -0,180 | **0,009** |
| Potassium (mg) | 0,117 | 0,092 |
| Selenium (mg) | 0,098 | 0,155 |

TEI: Total Energy Intake; SFA: Saturated Fatty Acids; MUFA: Monounsaturated Fatty Acids; PUFA: Polyunsaturated Fatty Acid

**Supplementary Figure 1.** Completion rates for EMA dietary questions over time stratified by sex and BMI category. Values represent mean completion rates (%) ± standard error (SE) across study months.
